# Green mycosynthesis of ZnO nanoparticles enhances antifungal defense against *Fusarium equiseti* through metabolic and gene expression modulation

**DOI:** 10.1186/s11671-026-04429-5

**Published:** 2026-01-30

**Authors:** EL-Sayed M. El-Morsy, Yomna S. Elmalahy, Elsayed E. Hafez

**Affiliations:** 1 Botany and Microbiology Department, Faculty of Science, Damietta University, New Damietta, 34517 Egypt; 2 Plant Protection and Bimolecular Diagnosis Department, (Arid Lands Cultivation Research Institute), City of Scientific Research and Technological Applications, New Borg El-Arab, Egypt

**Keywords:** *Fusarium equiseti*, Zinc oxide nanoparticles, *Trichoderma asperellum*, Green synthesis, Antifungal activity, GC–MS, qRT–PCR

## Abstract

The soil borne phytopathogen *Fusarium equiseti* poses a significant threat to global crop production, with limited sustainable control options. In this study, zinc oxide nanoparticles (ZnO-NPs) were green-synthesized using the extracellular filtrate of *Trichoderma asperellum* and characterized by SEM, XRD, FTIR, DLS, and Zeta potential apparatuses. The biosynthesized ZnO-NPs exhibited a quasi-spherical morphology with a size range of 13–19 nm, high crystallinity, and a stable negative zeta potential (− 27 mV). FTIR confirmed successful ZnO-NPs formation through the characteristic absorption band at 599 cm^−1^. Antifungal assays revealed a concentration dependent inhibition of mycelial growth in two examined strains of *F. equiseti*, achieving maximum suppression of 72.41 ± 0.01 and 73.41 ± 1.16% at 5 mg mL^−1^, comparable to the commercial fungicide Propiconazole (5 mg mL^−1^) with inhibition rates reaching 82.75 ± 0.75% for *F. equiseti* st.1 and 85.49 ± 0.68% for *F. equiseti* st.2. GC–MS profiling of the treated fungal filtrates indicated that, ZnO-NPs induced distinct metabolic alterations, including the appearance of stress associated metabolites such as phomenone, alongside modulation of membrane active glycol ethers when compared with the non-treated fungi. qRT–PCR analysis showed that ZnO-NPs selectively enhanced the expression of defense related genes (PR2, PPO, PR5, and PR8) in two strain of *F. equiseti*, while response efficacy was differed between the two strains. These findings demonstrate that mycosynthesized ZnO-NPs exert potent antifungal activity through combined physical disruption and biochemical interference, offering a promising eco-friendly alternative to synthetic fungicides for sustainable crop protection.

**Supplementary Information:**

The online version contains supplementary material available at 10.1186/s11671-026-04429-5.

## Introduction

The genus *Fusarium* includes numerous species responsible for infections in both humans and plants [1]. Comprising over 300 phylogenetically distinct species, these filamentous fungi rank among the top ten most devastating plant pathogens worldwide [2–4]. Through mycelium or developing spores, the fungus enters the tomato plant’s xylem vessel and blocks the transport of nutrients [5]. When plants transpire more than they can carry, their stomata close, the plant will wilt and eventually die as a result of increased transpiration and decreased nutrient transfer [6, 7]. Along with the well-known *Fusarium oxysporum* species that cause wilt and root rot in a variety of economically important crops, other *Fusarium* species that cause wilt in various vegetables, such as bell paper, chili, cauliflower, sweet pepper, onion, potato, tomato, and many more, are constantly evolving. Examples of these species are *F. solani* and *F. equiseti* [2, 8]. *Fusarium equiseti* is increasingly associated with wilt diseases in tomatoes and other vegetables, contributing to severe postharvest losses. *Fusarium* wilt represents a major economic threat to tomato production worldwide, with yield reductions of 30–40% in India and up to 80% under favorable conditions, while regional losses range from 25–55% across different states [9]. In Egypt, *Fusarium oxysporum* has caused up to 67% yield losses in tomato production [10]. Globally, *Fusarium* induced wilt diseases impose economic costs amounting to hundreds of millions of dollars annually due to reduced yields, poor fruit quality, and high management expenses[11]. Because the pathogen is soil and seed borne and can persist for a long time as resting spores in soil (chlamydospores) and mycelium in contaminated plant debris, managing illness in the field is challenging [12]. Conventional disease control methods predominantly rely on synthetic fungicides. However, their widespread use has led to environmental contamination, health hazards, and the emergence of fungicide resistant *Fusarium* strains, including documented resistance to benzimidazoles and dicarboximides [13–16].

Moreover, these fungicides persist in soil and water, disrupt microbial activity, reduce biodiversity, and adversely affect non target organisms [17]. while microbial biofungicides and biologically synthesized nanoparticles offer safer, eco-friendly alternatives. Although they face challenges like shorter shelf life and limited availability, these bio-based options support soil health, reduce resistance risks, and can be integrated with lower chemical doses to improve efficacy and minimize toxicity [17, 18].

Traditional physical and chemical nanoparticle synthesis methods often involve hazardous reagents, high energy consumption, and toxic byproducts. In contrast, green synthesis, which employs biological resources such as plant extracts, bacteria, or fungi offers a safer, more sustainable alternative. This approach aligns with the principles of green chemistry, focusing on waste reduction, energy efficiency, use of renewable feed stocks, and minimized toxicity [19–21].

Nano materials can be used as a substitution fungicide which able to reduce plant diseases and improve development by limiting nutrient loss. Bacteria, algae, plants, diatoms, and fungus are called NP manufacturers due to their reducing and stabilizing properties [22, 23]. Fungi especially *Trichoderma* are particularly interesting because of their rapid mycelia growth, which provides greater surface area, easy biomass management, and large scale production [19, 21]. In Additionally, their ability to produce significant amounts of proteins could boost nanoparticle manufacturing productivity [7, 8, 10]. Metal and metal oxide nanoparticles have demonstrated promising antibacterial and antifungal properties, making them valuable in biosensing, catalysis, optoelectronics, and agricultural biotechnology [24, 25]. In parallel with the growth of nanotechnology, environmental concerns such as climate change, pollution, and resource depletion have led to increased demand for green and sustainable technologies[26].

Given these challenges, the development of eco-friendly antifungal agents is urgently needed. Metal oxide nanoparticles such as ZnO, MgO, and CaO, known for their high surface reactivity and distinctive crystal structures, present a promising solution [27–29]. Among these nanomaterials, ZnO-NPs have attracted considerable interest due to their potent biological activity, low cost, chemical stability, and biocompatibility compared to other metal NPs such as silver [30, 31]. Moreover, ZnO is a Generally Recognized As Safe (GRAS) material, and both bulk and nano forms of ZnO have shown effectiveness against a broad spectrum of bacterial and fungal pathogens [32, 33]. ZnO-NPs, in particular, offer a combination of antifungal efficacy, biocompatibility, and thermal stability, making them ideal candidates for agricultural applications [34, 35]. Biological entities synthesize ZnO-NPs via various metabolites such as proteins, enzymes, and other biomolecules that serve as reducing, capping, and stabilizing agents. ZnO-NPs vary in shape, size, dispersity, and stability according on secreted metabolites [36]. Enzymatic mechanisms especially those involving NADH dependent reductases drive extracellular reduction of Zn^2^⁺ ions into elemental Zn, leading to nanoparticle nucleation and growth. Such enzymes, along with peptides and amino acids such as cysteine, tyrosine, tryptophan, also function as capping molecules to stabilize particles and prevent aggregation [37, 38]. Zinc is an essential trace element found in all bodily tissues and enzyme systems. At the Nano scale, ZnO-NPs are easily adsorbed due to their small size and can be utilized as food additives due to their FDA recommendation as a safe substance [34, 39].

Nanoparticles derived from biological sources particularly green-synthesized ZnO‑NPs demonstrate remarkably high antifungal activity against *Fusarium equiseti*. A recent study using tea leaf extract reported that ZnO‑NPs at concentrations of 750–1200 ppm inhibited *F. equiseti* growth by 77.6–85.1%. while microscopy confirmed severe hyphal deformation and mycelial disruption. [40–42]. Similarly, ZnO‑NPs synthesized via endophytic fungi or *Penicillium expansum* were effective not only in vitro, but also in vivo: treated eggplants infected with *Fusarium oxysporum* showed 75% disease suppression, significant improvement in growth parameters (plant biomass, chlorophyll content, protein levels) [19, 43].

Comparative studies have demonstrated that myco-synthesized ZnO-NPs, particularly those produced by fungi such as *Trichoderma harzianum*, exhibit significantly higher antifungal potency against *Fusarium oxysporum* at lower doses compared to bacteria derived counterparts. In one recent study, *Trichoderma* mediated ZnO-NPs (~ 29 nm) inhibited *Alternaria brassicae* growth by 91.5% at 200 μg/mL, outperforming chemically synthesized ZnO-NPs (79.6%) and conventional fungicide (82.96%) at the same concentration through mechanisms involving ROS generation, membrane damage, and enhanced antioxidant enzyme activity [19, 44]. Similarly, ZnO-NPs biosynthesized using *T. harzianum* showed complete in vitro inhibition of several soil-borne pathogens including *Fusarium sp.* and *Rhizoctonia solani*, with markedly greater efficacy than standard chemical fungicides, both in laboratory assays and greenhouse [33, 45]. This superior performance of mycogenic ZnO-NPs over bacteria derived nanoparticles is likely due to finer particle size, optimized morphology, and capping with fungal metabolites that enhance surface chemistry, stability, and bioactivity. These characteristics result in lower MICs (minimal inhibitory concentrations) and stronger antifungal effects, confirming that green synthesis via fungal biomass produces ZnO-NPs with optimized structure and function for sustainable crop protection [46, 47].

This study focuses on the green synthesis of ZnO-NPs using *Trichoderma asperellum* filtrate as reducing biological substrates. Evaluation the antifungal activity of the biosynthesized ZnO-NPs against two strains of *Fusarium equiseti*. In addition, the effects of the ZnO-NPs on the two phytopathognic on the physiological and molecular levels. Finally, explore the potential of ZnO-NPs as a sustainable alternative to conventional fungicides, thereby contributing to safer agricultural practices and reduced environmental impact.

## Materials and methods

### Fungal strains collection and identification

The two pathogenic fungi as *F.equiseti st.1* and *F.equiseti st.2* were isolated from tomato plant (GS12) in Damietta city, Egypt. And identified as *Fusarium equiseti* st.1 ON533655,* Fusarium equiseti* Fusarium equiseti st.2 ON533656. And this have different morphological character and pathogenicity,their identification character were reported in our pervious study [48]. *Trichoderma asperellum* OP889678 was kindly provided from Dr. Elsayed E. Hafez lab,Plant Protection and Bimolecular Diagnosis Department, Arid Lands Cultivation Research Institute, City of Scientific Research and Technological Applications, New Borg El-Arab, Egypt.

### Preparation of zinc oxide nanoparticles

ZnO-NPs were prepared according to the method with minor modification [49]. A 7 day old culture of *Trichoderma asperellum*., 1 cm diameter mycelia disc was inoculated into 100 mL of potato dextrose broth and incubated at 25 °C for 5 days*.* The culture was then filtered through Whatman No. 1 filter paper. Then 1 g of zinc sulphate (ZnSO_4_. 7H_2_O) (Alfa chemicals, Egypt) was added to 100 ml of the fungal culture filtrate and dissolved well, then one drop by drop (0.1 M) of NaOH solution was added until white dispersed nanoparticles appeared and then incubated in the shaking incubator for 24 h at 40 °C. The mixture was centrifuged at 4000 rpm for 20 min and then the precipitate was dried at 50 °C in oven.

### Characterization techniques

Fourier transform infrared (FTIR) spectra were recorded using KBr pellets on a JASCO FT/IR-4100 spectrometer **(**Japan Spectroscopic Company, Japan). The surface morphology of the ZnO-NPs was examined using a scanning electron microscope (SEM, JEOL JSM-IT200, JEOL Ltd.,). Zeta potential measurements were performed with a Malvern Zetasizer Nano-ZS90 (Malvern, UK). X-ray diffraction (XRD, (Siemens AG, Germany)) patterns were obtained using a Siemens D-500 diffract meter.

### Antifungal activity

ZnO-NPs were evaluated as antifungal compound against two strains of *F. equiseti* by agar well diffusion method. Potato dextrose agar (PDA) medium was prepared and supplemented with different concentrations of ZnO-NPs (1, 2, 3, 4, and 5 mg/mL). Each Petridish was inoculated at the center with a 1 cm diameter mycelia disk obtained from a 7 day old culture of *F. equiseti*. [50]. All Petri dishes were incubated at 25 °C for 7 days, and daily measurements of radial colony growth were taken until the fastest growing colony approached the plate’s edge. All of the treatments had three replications, and the experiment was carried out twice. Using Vincent’s formula, the percent inhibition rate of the pathogen’s mycelia was calculated by comparing the treatment plates with commercial fungicide and the control plate (without nanoparticles).

1 $$\% Inhibition rate= \frac{{M}_{C}- {M}_{t}}{{M}_{c}} \times 100$$

While M_c_ and M_t_ are the mycelia growth in control and the mycelia growth in treatment with different conc of nanoparticles, respectively.

### Extraction of fungal active compounds existed in their culture filtrate

Both strains of *Fusarium equiseti* were cultured in potato dextrose broth medium and treated with the optimal concentration of ZnO-NPs and as well as the commercial fungicide(Propiconazole (25%)). The cultures were incubated for seven days at 25 °C. Afterward, the fungal culture were centrifuged at 4000 rpm for 10 min. The active compounds were then separated by mixing the filtrate with an equal volume of ethyl acetate using a separation funnel. The organic phase was collected, mixed with anhydrous sodium sulfate for drying. To prevent degradation of bioactive compounds, the drying procedure was done at ambient temperature [51, 52].

### GC–MS analysis of fungal filtrate extract

The extract solid material was dissolved in chloroform and was subjected analysis by using GC–MS (GAS CHROMTOGRAPHY GC-2010 PLUS,SHIMADZU). The column oven temperature was kept at 50 °C at first, then increased at a rate of 5 °C per minute to 230 °C and held for 2 min before being increased to the final temperature of 290 °C and held for 2 min. The sample (1 µl) was injected at 250 °C with helium as a carrier. The names, molecular weights, and chemical structures of the discovered compounds were also determined [51].

### Quantitative real-time PCR (qRT-PCR)

To evaluate the expression of defense-related genes in the treated *Fusarium equiseti* strains with ZnO-NPs and a commercial fungicide. Total RNA was extracted from both treated and untreated fungal cultures. To synthesize complementary DNA (cDNA), 3 μL of total RNA was mixed with 5 μL of oligo (dT) primer (10 pmol/μL), 2.5 μL of dNTPs (10 mM), 2.5 μL of 10 × reverse transcriptase buffer, 0.3 μL of Reverse Transcriptase Enzyme, and 6.7 μL of sterile distilled water in a 20 μL reaction mixture. The reverse transcription reaction was performed in a Thermal Cycler with the following program: 37 °C for 1 h and 65 °C for 10 min to inactivate the enzyme. The synthesized cDNA was kept at -20 °C for future analysis. SYBR Green chemistry was used for quantitative real-time PCR (qRT-PCR) with a final reaction volume of 20 μL. Ten microlitres of SYBR Green Master Mix (Fermentas, USA), one microlitre of forward primer (10 pmol/μL), one microlitre of reverse primer (10 pmol/μL), one microlitre of cDNA (50 ng), and seven microlitres of sterile distilled water were included in each reaction. Initial denaturation at 95 °C for 10 min, 45 cycles of denaturation at 95 °C for 10 s, annealing at 60 °C for 20 s, and extension at 72 °C for 20 s were the cycling conditions used for the amplification, which was carried out on a Rotor-Gene 6000 system (Qiagen, USA). Data on fluorescence was gathered during the expansion stage. Livak and Schmittgen’s 2^–ΔΔCT technique were used to analyze relative gene expression. The housekeeping gene β-actin was used to normalize the expression levels of the genes linked to pathogenesis (PR2, PPO, PR5, and PR8). To evaluate how antifungal treatments affected gene regulation, each reaction was carried out in triplicate, and mean results were computed [52].

### Statistical analysis

All experiments were performed in triplicate to ensure reproducibility. Data are presented as mean values ± standard deviation (SD), and error bars indicate the SD.

## Results

### Characterization of ZnO-nanoparticles

ZnO-NPs were synthesized utilizing cell-free extracellular filtrate from a 7-day-old *Trichoderma asperellum* filtrate. The biological synthesized ZnO-NPs has shown promising results in terms of performance, eco-friendliness, cost-effectiveness, and increased productivity [53]. However, the current green synthesis of NPs is simple, non-toxic, and does not require sintering.

The biosynthesized of ZnO-NPs were characterized using DLS, SEM, XRD, FTIR analysis, GS-MS spectrometer and PCR analysis.

### Dynamic light scattering (DLS) and zeta potential analyses

**Fig. 1:**
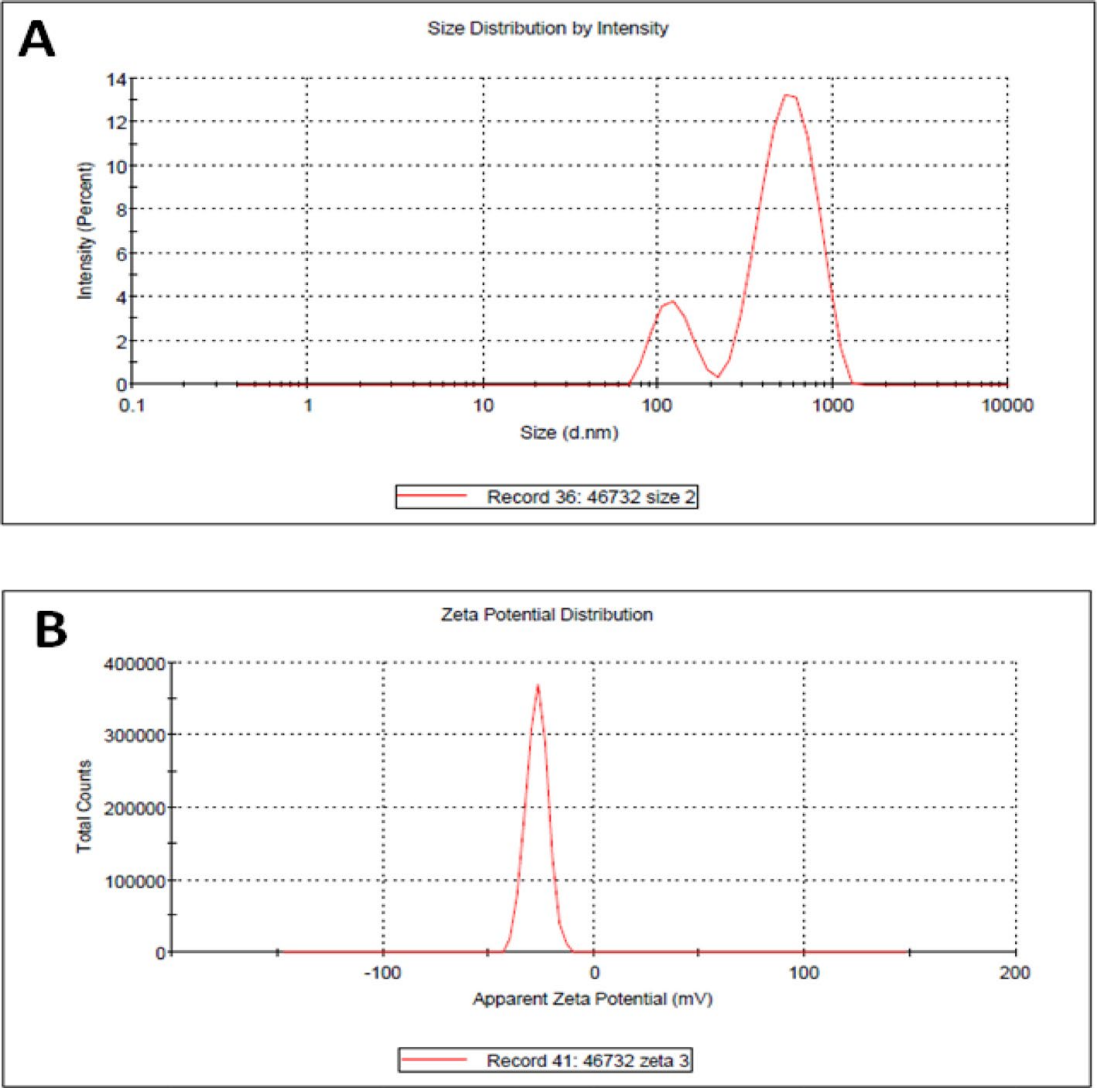
Particle size distribution and zeta potential of ZnO-NPs obtained from DLS

DLS is a commonly used technique for detecting particle size distribution in colloid solutions based on intensity. ZnO-NPs were found to be poly-dispersed, with average diameters of 581.9 nm (83.4%) and 124.8 nm (16.6%). DLS measurements of particle diameters differed from SEM measurements due to factors such as capping proteins and enzymes around the particles [54, 55]. ZnO-NPs have a negative zeta potential of -27 mV, indicating their stability (see Fig. 1). Particles with zeta potentials above + 30 mV or below − 30 mV are considered stable. ZnO-NPs synthesized via biosynthesis have a zeta potential of − 27 mV and are highly stable. The presence of negatively charged groups on the NPs surface was associated with a significant negative zeta potential. Protein and flavonoids in the leaf extract may be responsible for metal ion reduction and NPs stabilization.

### SEM analysis of ZnO-NPs

**Fig. 2:**
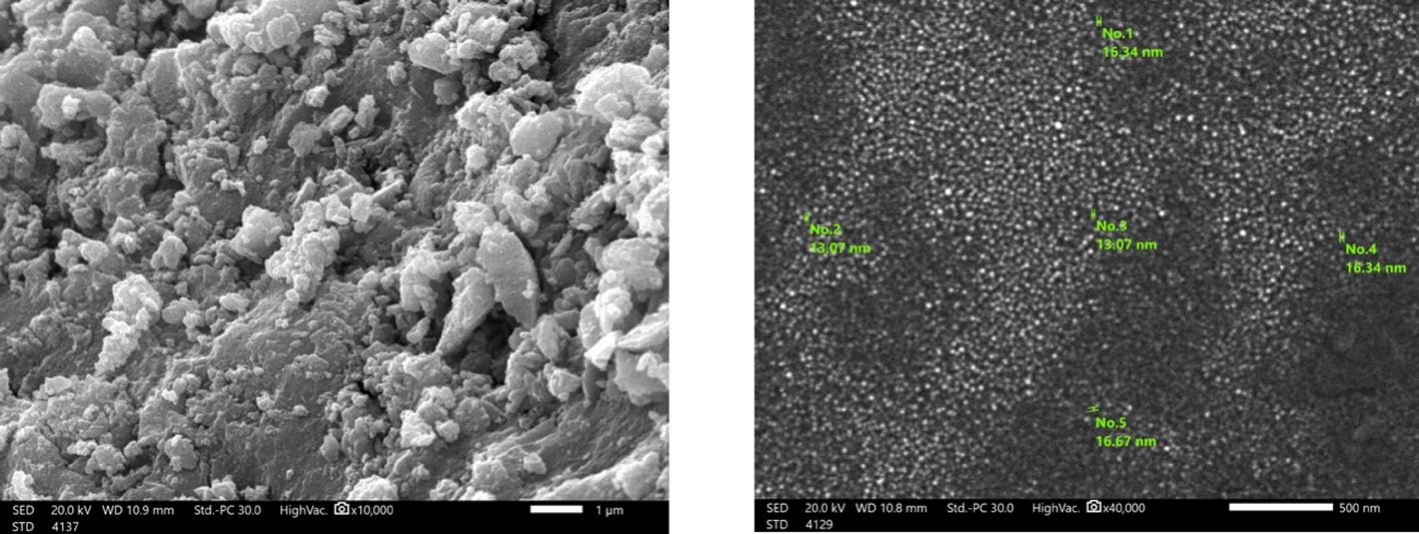
Scanning electron microscope of biosynthesized ZnO-NPs

Scanning Electron Microscopy (SEM) is a high resolution surface imaging technique that uses an electron beam to obtain information about nanostructures and other materials on a microscopic scale. SEM pictures are useful for obtaining surface topological information about diverse nano objects based on the electron density of the surface due to their higher magnification and field depth. SEM pictures show that the particles are virtually spherical. The particles clumped together to form a sponge like collection of particles. This agglomeration could be caused by densification, which results in a small space between particles [56, 57] The SEM analysis confirms that the green synthesis process produced spherical ZnO-NPs with a narrow size distribution (13–19 nm) as in Fig. 2.

### X-ray diffraction (XRD) analysis

**Fig. 3:**
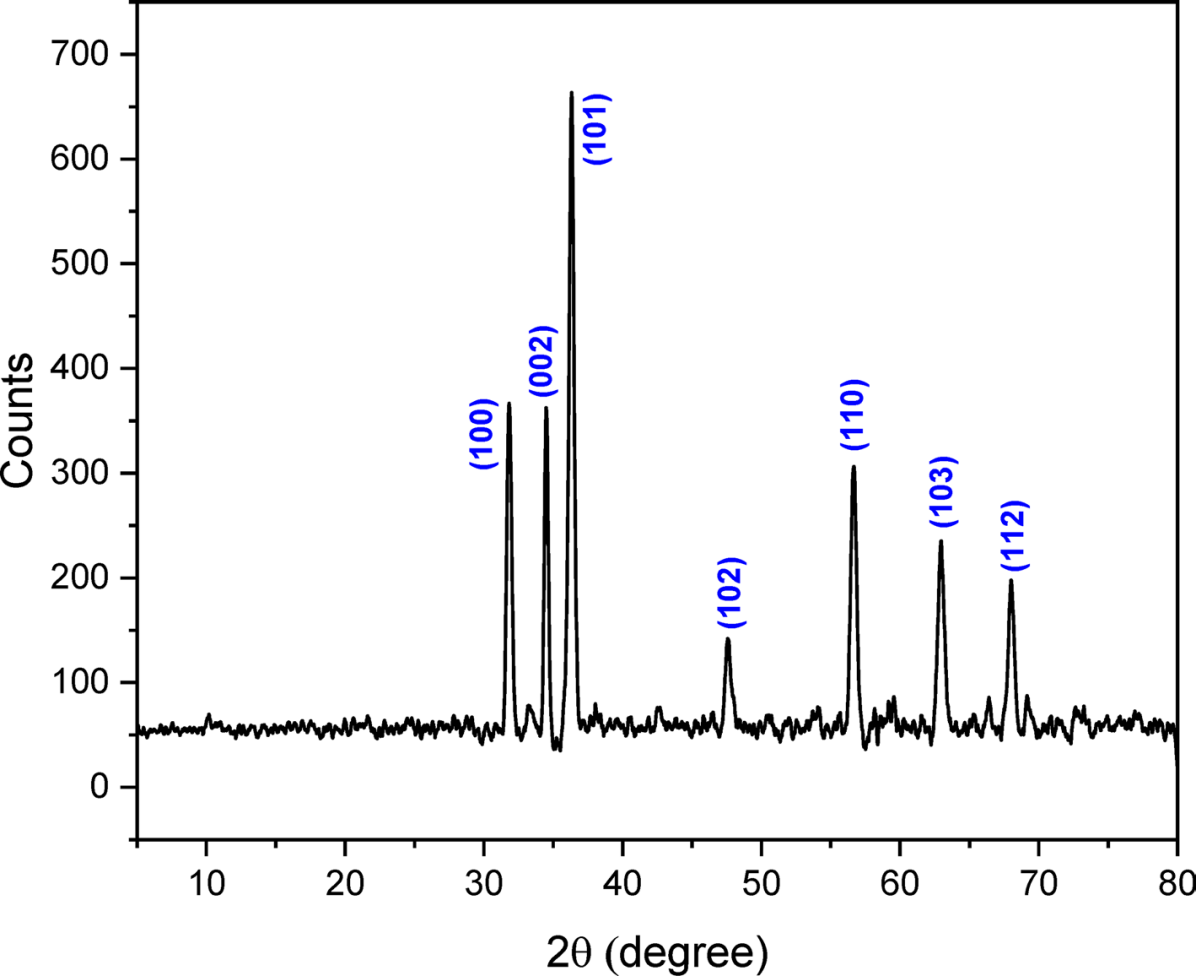
XRD analysis of biosynthesized ZnO-NPs

**Fig. 4:**
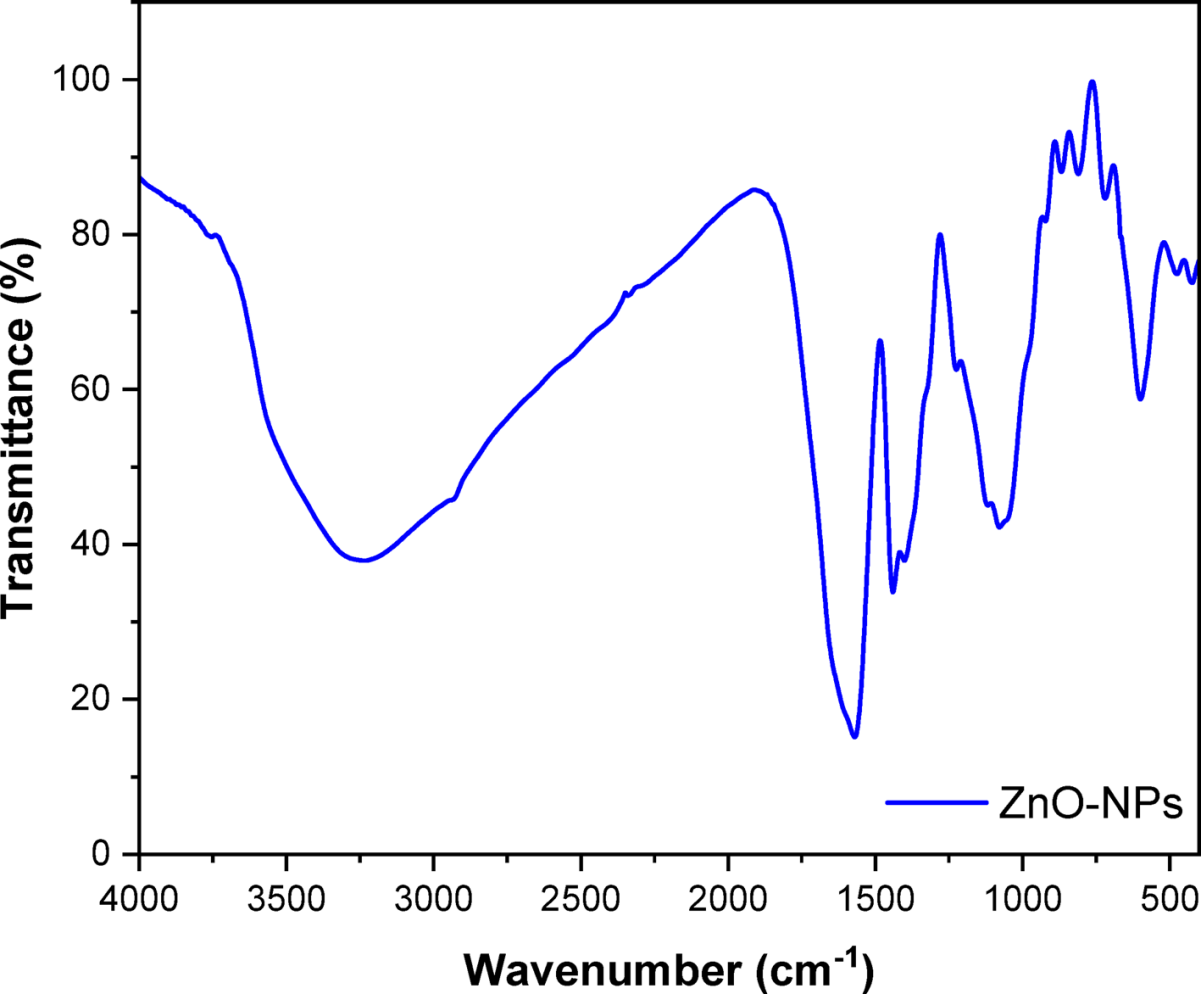
FTIR analysis of biosynthesized ZnO-NPs

The crystalline structure of the biosynthesized ZnO-NPs was confirmed via X-ray diffraction (XRD) analysis, with diffraction patterns corresponding closely to the hexagonal phase of ZnO, as matched with the standard JCPDS card No. 01–089-0510. The diffraction spectrum (Fig. 3) displays prominent peaks at 2θ values of 31.8°, 34.4°, 36.3°, 47.5°, 56.6°, 62.8°, and 67.9**°**, which are indexed to the crystal planes (100), (002), (101), (102), (110), (103), and (112), respectively. This confirms the successful formation of hexagonally structured ZnO-NPs without secondary impurities or phases. (Fig. 4).

Moreover, the crystallite size of ZnO-NPs was determined from XRD data using Scherrer equation.

$$ D = \frac{K\lambda }{{\beta \cos \theta }} $$

D represents crystallite size (nm), K is the Scherrer constant (0.89), λ is the wavelength of the X-ray source (0.15406 nm), β is the full width at half maximum (FWHM), and θ is the peak location. The crystallite size was found to be 54.22 nm, indicating the formation of ultrafine crystalline particles with potentially enhanced surface reactivity and antimicrobial activity. These results align with previous studies demonstrating that green-synthesized ZnO-NPs exhibit high crystallinity and purity in the hexagonal phase, suitable for biological and agricultural applications [58].

### Fourier transform infrared spectroscopy (FTIR)

FTIR is employed as a confirmatory technique for nanoparticle creation and delivers an impression of the vibrational and rotational modes of the present molecules, therefore it helps to identify the functional and potential phytochemical compounds involved in the reduction and stabilization of ZnO-NPs [59]. The broad absorption peak at 3235 cm^−1^ is associated with NH, which overlaps with a stretched OH band [60]. The creation of intramolecular and intermolecular hydrogen bonding could account for the broadness of this peak [61]. The low-intensity peak at 3000 cm^−1^ is associated with the stretching CH_2_ of asymmetric and symmetric carbohydrates and/or lipids [62], whereas the band at 1570 cm − 1 corresponds to the stretching C=O vibration of proteins. Proteins’ absorption waves of CH_2_ or CH_3_ cause vibration bending of the C-H at 1440 cm^−1^ [61]. The band at 1400 cm^−1^ corresponds to the C-N stretching bond of amino acids, while the band at 1078 cm^−1^ is related to the C–O–C ether of polysaccharides [60, 61]. The absorption band at 599 cm − 1 confirmed the successful production of ZnO-NPs. The FTIR study of green synthesized ZnO-NPs revealed the ZnO absorption band at wavelengths 475 cm − 1, 423 cm − 1, and in the range 400 to 500 cm − 1 as shown in Fig. 1 [63].

### Effect of ZnO-NPs on mycelium growth of *F. equiseti*

**Fig. 5:**
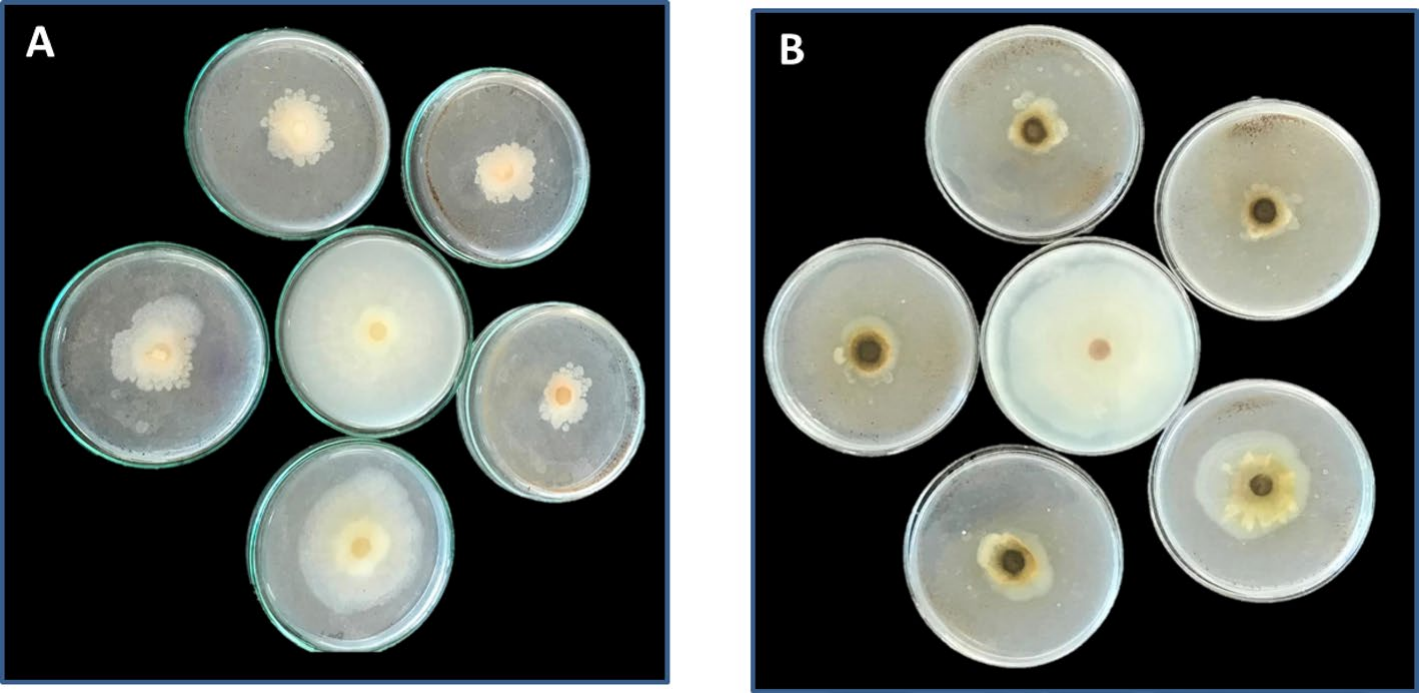
Determination of the effect ZnO-NPs on mycelium radial growth of the two strains A as *F. equiseti* st1 and B as *F. equiseti* st2

The studies were conducted in PDA media with ZnO-NPs and commercial fungicide Propiconazole with doses ranging from 1 to 5 mg/ml and incubated at 25 °C for 7 days. Table 1 and Fig. 5 show how ZnO-NPs affect mycelium radial growth in two strains of *F. equiseti.* Increasing the concentration of NPs results in higher inhibition percentages for both *F. equiseti* st.1 and *F. equiseti* st.2. At a dose of 5 mg/ml of ZnO-NPs, *F. equiseti* st.1 and *F. equiseti* st.2 showed maximum inhibition rates of 72.41 ± 0.01 and 73.41 ± 1.16% respectively. In comparison, Propiconazole displayed consistently higher antifungal activity across all concentrations, with inhibition rates reaching 82.75 ± 0.75% for *F. equiseti* st.1 and 85.49 ± 0.68% for *F. equiseti* st.2 at 5 mg/ml. These findings highlight that while ZnO-NPs possess strong antifungal potential, their efficacy is still slightly lower than that of the commercial fungicide. Nevertheless, ZnO-NPs offer a promising eco-friendly alternative due to their biocompatibility, potential for green synthesis, and reduced environmental toxicity compared to synthetic fungicides.

**Table 1:** Inhibition rates (mean ± SD) of *Fusarium equiseti* strains (st.1 and st.2) treated with different concentrations (1–5 mg/ml) of ZnO-NPs and commercial fungicide (Propiconazole)

Concentration (mg/ml)	ZnO-NPs *F. equiseti* st.1	ZnO-NPs *F. equiseti* st.2	Fungicide *F. equiseti* st.1	Fungicide *F. equiseti* st.2
1	28.35 ± 1.39	20.39 ± 8.20	71.97 ± 1.50	78.82 ± 0.00
2	52.87 ± 4.22	47.28 ± 8.07	74.98 ± 1.50	80.97 ± 0.34
3	63.73 ± 0.79	60.50 ± 2.93	80.59 ± 0.00	82.70 ± 0.96
4	66.79 ± 0.44	69.57 ± 0.00	81.88 ± 0.00	84.58 ± 0.20
5	72.41 ± 0.01	73.41 ± 1.16	82.75 ± 0.75	85.49 ± 0.68

### GC–MS analysis of the two phytopathogenic fungal filtrate extract

GC–MS analysis of the fungal filtrate extracts from *Fusarium equiseti* st.1 and st.2, both untreated (control) and treated with ZnO-NPs and commercial fungicide, revealed significant differences in their chemical profiles. The untreated *F.equiseti* st.1 contained a wide range of bioactive compounds, including 1-dodecanol, 1-tetradecanol, 1-hexadecanol, ethanol, 2-(dodecyloxy)-, diethylene and triethylene glycol monododecyl ethers, bis(2-ethylhexyl) phthalate, n-nonadecanol-1, and various other long-chain fatty alcohols and ethers. Similarly, the untreated *F. equiseti* st.2 was rich in 1-dodecanol, 1-hexadecanol, diethylene glycol monododecyl ether, triethylene glycol monododecyl ether, and tetraethylene glycol monododecyl ether, compounds known to be associated with membrane structure, lipid metabolism, and stress-related surfactant activity. These metabolites collectively play essential roles in fungal cell membrane integrity, detoxification processes, and stress response pathways.

*When treated with ZnO-NPs*, *F.equiseti* st.1 exhibited retention of key fatty alcohols, including 1-dodecanol, 1-tetradecanol, and 1-hexadecanol. However, a reduction in the abundance of some other fatty alcohols, such as 1-heptacosanol and 2-methylpentacosane, was observed. Notably, there was a marked increase in ethoxylated alcohols, including diethylene glycol monododecyl ether, triethylene glycol monododecyl ether, tetraethylene glycol monododecyl ether, and heptaethylene glycol monododecyl ether. Among these, triethylene glycol monododecyl ether increased from 17.36% to 19.67%, while tetraethylene glycol monododecyl ether rose from 15.99% to 16.57%, indicating a stress-induced upregulation of membrane-active glycol ethers. In *F.equiseti* st.2, ZnO-NPs treatment also preserved high levels of triethylene and tetraethylene glycol ethers, suggesting a sustained stress response. In contrast, levels of 1-hexadecanol and 1-dodecanol were reduced, implying disruption of membrane lipid synthesis. Importantly, Phomenone, a metabolite associated with oxidative stress or apoptosis, was detected only in the ZnO-NPs treated sample, further confirming the nanoparticle-induced biochemical stress on the fungal cells.

*When treated with commercial fungicide*, *F.equiseti* st.1 occur increase in triethylene glycol monododecyl ether (18.32%), along with high levels of tetraethylene glycol monododecyl ether (13.82%). Additionally, the presence of Tilt (isomer 2)—a known fungicide marker—and the appearance of 3,7,11,15-tetramethylhexadecane-1,2,3-triol triacetate, a byproduct of lipid metabolism, were noted. There was also an elevation in bis(2-ethylhexyl) phthalate, which may reflect increased oxidative or membrane stress. In, *F.equiseti* st.2, pest exposure led to a rise in diethylene glycol monododecyl ether and the appearance of Tilt (isomer 2), which could indicate either pesticide residue or a defensive metabolic response. However, Phomenone, a metabolite associated with oxidative stress and cell death, was absent in this treatment, suggesting a weaker oxidative stress response compared to ZnO-NPs exposure.

Overall, ZnO-NPs induced more pronounced biochemical alterations, including the production of unique stress metabolites and membrane disruptive compounds, indicating a stronger antifungal mechanism than that observed under chemical fungicide. These findings demonstrate the value of GC–MS profiling in evaluating antifungal treatments and support the application of metal based nanomaterials.

To better visualize the biochemical differences observed between control and treated fungal samples, the major GC–MS peaks were compiled and compared in structured tables for *Fusarium equiseti* st.1 and st.2. as shown in Tables (2 and 3). These highlight the retention time (RT) and relative abundance (%) of key metabolites detected in the fungal filtrates under untreated conditions and after exposure either to ZnO-NPs or commercial fungicide. These metabolites include long-chain alcohols, ethoxylated surfactants, phenolic antioxidants, and specific chemical makers such as Tilt (isomer 2), which is characteristic of fungicide residues. The inclusion of stress-associated compounds like Phomenone and 13-docosenamide, observed exclusively in ZnO-NPs, further supports the unique biochemical impact of nanomaterial exposure. Through this comparative tabulation, the enhanced antifungal effects of ZnO-NPs evident in both compound diversity and abundance shifts are made clear, underscoring their potential as effective and alternative antifungal agents.

### Quantitative real-time PCR (qRT-PC)

Real-time PCR was employed to quantify the relative mRNA levels of four related protein genes in two strains of *F. equiseti* and following treatment with ZnO-NPs and a commercial fungicide. The analyzed defense genes of *F. equiseti* st.1 include β-1, 3-glucanases (PR2), Polyphenol Oxidase (PPO), thaumatin like proteins (PR5) and Class III Chitinase (PR8) under different treatments control, ZnO-NPs, and fungicide as in Fig. 5. For PR2, expression levels remained relatively low and comparable among the three treatments, with a slight increase under fungicide application. PPO expression was markedly upregulated in both ZnO-NPs and fungicide treated samples compared to the control, indicating strong induction by both treatments. PR5 expression levels were similar in the control and ZnO-NPs groups, but substantially reduced under fungicide treatment. In contrast, PR8 expression showed a notable increase with ZnO-NPs compared to the control, while fungicide treatment caused a dramatic reduction to near baseline levels. These results suggest that ZnO-NPs selectively enhanced PPO and PR8 expression, whereas fungicide treatment strongly induced PPO but suppressed PR5 and PR8 expression. (Tables 2 and 3).

**Table 2:** Comparative Table of Major GC–MS Peaks in Control, ZnO-NP, and Fungicide Treatments of *Fusarium equiseti* st.1

Compound Name	RT (min)	Control (%)	ZnO-NPs (%)	Fungicide (%)
Triethylene glycol monododecyl ether	~ 37.3–44.4	23.0%	24.85%	22.96%
Tetraethylene glycol monododecyl ether	~ 42.0–45.6	17.89%	18.91%	15.55%
Diethylene glycol monododecyl ether	~ 32.3–43.2	19.81%	18.78%	22.15%
Heptaethylene glycol monododecyl ether	~ 46.8–50.9	6.55%	9.09%	8.15%
Ethanol, 2-(dodecyloxy)-	~ 26.6	5.51%	5.45%	6.07%
Ethanol, 2-(hexadecyloxy)-	~ 30.8–34.7	3.77%	3.27%	3.52%
Tilt (isomer 2) *(Fungicide Marker)*	~ 38.4–38.7	0.00%	0.00%	2.90%
1-Dodecanol	~ 20.9	1.18%	1.66%	2.02%
Bis(2-ethylhexyl) phthalate	~ 41.7	2.76%	3.28%	2.43%
2,4-Di-tert-butylphenol	~ 21.7	0.00%	0.00%	0.51%

**Table 3:** Comparative Table of Major GC–MS Peaks in Control, ZnO-NP, and Fungicide Treatments of *Fusarium equiseti* st.2

Compound	R.T. (min)	Control (%)	ZnO-NPs (%)	Fungicide (%)
Triethylene glycol monododecyl ether	37.34–47.88	19.97% (3 peaks)	19.65% (3 peaks)	18.98% (3 peaks)
Tetraethylene glycol monododecyl ether	42.01	16.13%	16.80%	13.53%
Diethylene glycol monododecyl ether	32.32–43.22	9.60% (2 peaks)	16.4% (4 peaks)	15.17% (2 peaks)
Heptaethylene glycol monododecyl ether	45.66–50.96	5.17% (3 peaks)	12.7% (4 peaks)	5.32% (3 peaks)
1-Dodecanol	20.91	1.59%	0.79%	0.80%
1-Hexadecanol	25.71	4.09%	1.67%	Not Detected
1-Tetradecanol	24.21	Not Detected	Not Detected	2.36%
n-Nonadecanol-1	30.05 & 34.01	3.03% (2 peaks)	3.60% (2 peaks)	5.58% (2 peaks)
2,4-Di-tert-butylphenol	19.24	1.01%	Not Detected	0.54%
Bis(2-ethylhexyl) phthalate	41.73	6.76%	4.76%	2.90%
Cyclononasiloxane, octadecamethyl-	38.57–43.33	4.62% (2 peaks)	3.21% (3 peaks)	1.71% (2 peaks)
Phomenone	37.08	Not Detected	0.80%	Not Detected
13-Docosenamide (Z)	38.81	Not Detected	0.70%	Not Detected
Tilt (isomer 2) (fungicide marker)	36.31 & 40.30	Not Detected	Not Detected	3.45% (2 peaks)

**Fig. 6:**
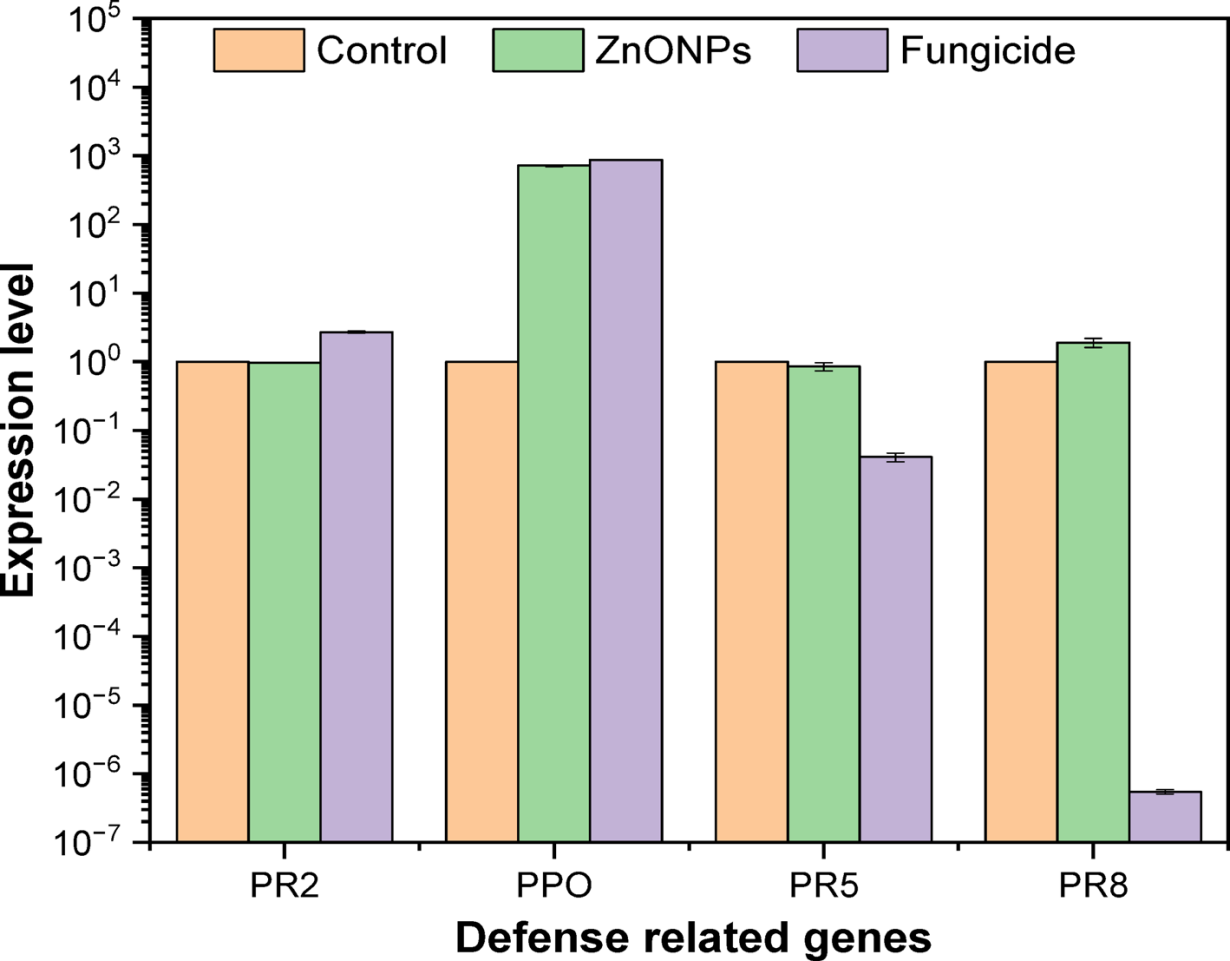
Histogram showing the quantitative expression levels of *PR2*, *PPO*, *PR5*, and *PR8* genes in *Fusarium equiseti* st.1, and following treatments with ZnO-NPs and a commercial fungicide

**Fig. 7:**
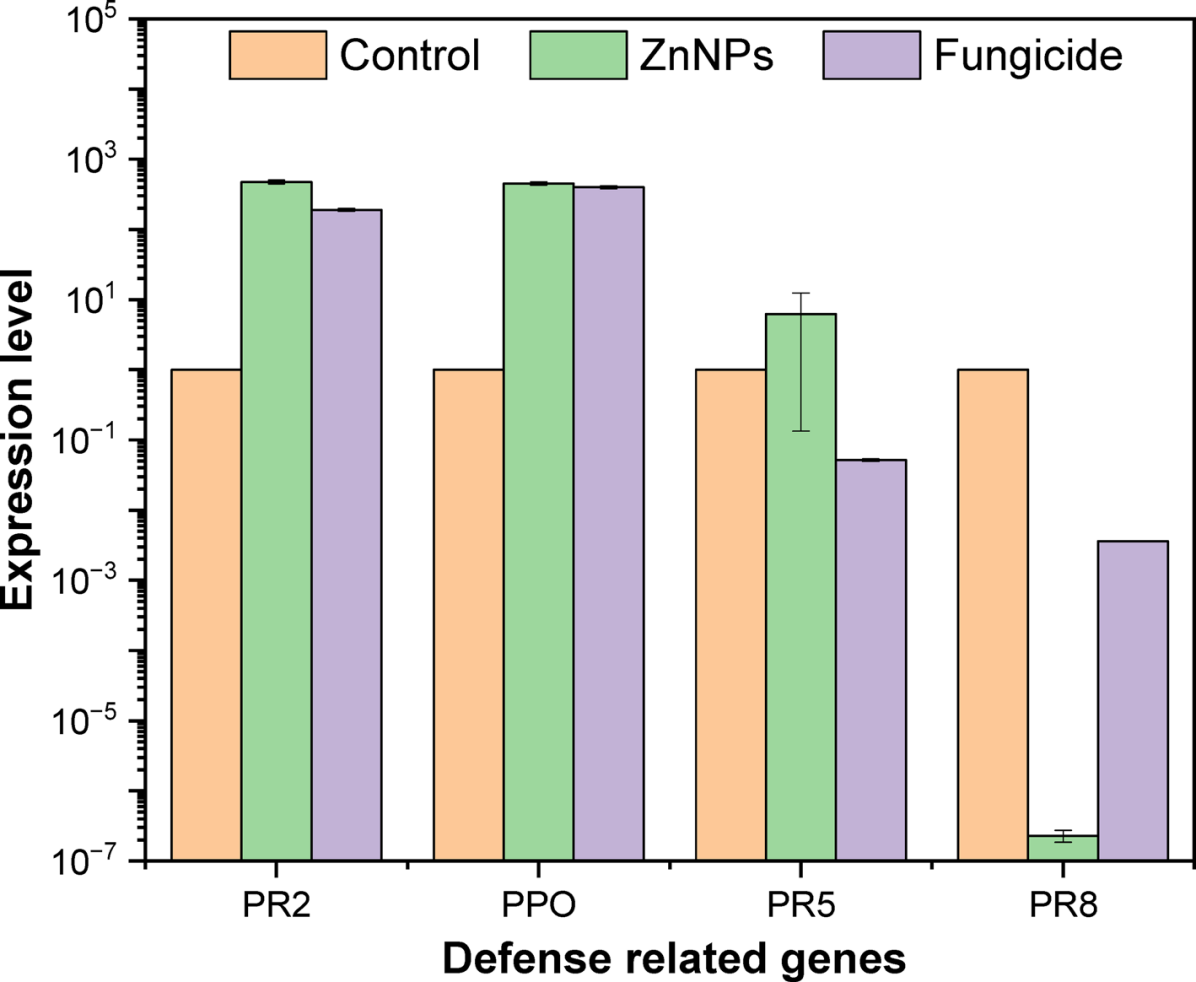
Histogram showing the quantitative expression levels of *PR2*, *PPO*, *PR5*, and *PR8* genes in *Fusarium equiseti* st.2, and following treatments with ZnO-NPs and a commercial fungicide

While the PCR analysis showed notable variations in the expression of PR2, PPO, PR5, and PR8 genes in *F. equiseti* st.2 across control, ZnO-NPs, and fungicide treatments as shown in Fig. 6. PR2 expression was markedly upregulated in both ZnO-NPs and fungicide treated samples compared to the control, with ZnO-NPs showing the highest induction. Similarly, PPO expression increased significantly under both ZnO-NPs and fungicide treatments, far exceeding control levels, indicating strong activation by these treatments. PR5 expression was also enhanced under ZnO-NPs compared to the control, whereas fungicide treatment led to a pronounced reduction. In contrast, PR8 expression remained similar to control levels in untreated samples, was drastically suppressed by ZnO-NPs to nearly undetectable levels, and showed a moderate increase under fungicide treatment. These results suggest that ZnO-NPs strongly stimulate PR2, PPO, and PR5 expression but inhibit PR8, while fungicides enhance PR2 and PPO expression yet suppress PR (Fig. 7).

## Discussion

*Fusarium equiseti* is a soil borne filamentous fungus widely distributed in temperate and subtropical regions, causing root rot, crown rot, wilt, and damping off in a wide variety of crops [64]. Besides its pathogenicity, *F. equiseti* produces several mycotoxins, including trichothecenes and zearalenone, which threaten food safety and animal health [65, 66]. Traditional management relies on fungicides and crop rotation, but concerns about resistance and environmental impact have accelerated research into nanomaterials such as biosynthesized ZnO-NPs as eco-friendly antifungal alternatives [67, 68].

In this study, ZnO-NPs synthesized via an eco-friendly and green method using *Trichoderma asperellum* demonstrated strong antifungal potential, achieving inhibition rates of 72.41 ± 0.01 and 73.41 ± 1.16 against *F. equiseti* St.1 and *F. equiseti* St.2, respectively, at a concentration of 5 mg/mL. SEM analysis revealed quasi spherical, clustered morphologies with average size equal to 13–19 nm [69]. In addition, zeta potential indicate the surface charge was − 27 mV, indicating moderate stability[70]. Meanwhile, XRD confirmed high crystallinity typical of biologically synthesized ZnO-NPs and these results was similar to the properties of other reported studies [71]. FTIR spectra indicated fungal proteins, polysaccharides, and phenolics acting as reducing and capping agents, which not only stabilized the nanoparticles but may also have enhanced their antimicrobial performance [19, 72]. Our GC–MS analysis revealed that untreated *Fusarium equiseti* cultures were dominated by glycol ethers and long chain alcohols, which are central to membrane integrity, detoxification, and cellular signaling [73]. Following ZnO-NPs treatment, moderate remodeling was observed, with elevated triethylene and tetraethylene glycol monododecyl ethers and the appearance of phomenone, a stress-related sesquiterpenoid, suggesting the activation of oxidative stress and membrane repair pathways. Similar metabolic adjustments have been reported in *Fusarium* sp exposed to ZnO-NPs or plant derived antifungals, where ROS induction and lipid remodeling serve as primary defense mechanisms [74]. By contrast, fungicide treatment caused more severe metabolic disruption, with fungicide specific compounds such as Tilt isomer 2 and methacrylic acid ester and reduced lipid diversity, consistent with earlier findings that chemical antifungals trigger direct suppression of *Fusarium* sp metabolism [73, 75]. Overall, these results align with recent metabolomics studies indicating that *Fusarium* sp adapts to nanoparticle stress through oxidative and membrane remodeling responses, whereas conventional fungicides induce immediate biochemical inhibition [64, 76]. qPCR analysis showed that ZnO-NPs and fungicides trigger distinct defense responses in *Fusarium equiseti* st.1 and st.2. In both isolates, PPO was markedly upregulated by ZnO-NPs and fungicide compared with control, highlighting its central role in oxidative stress response and defense activation. Similarly, PR2 expression was elevated, consistent with its function in glucan hydrolysis and cell wall defense [77, 78]. Interestingly, PR5 showed contrasting regulation: while moderately induced by ZnO-NPs in *F. equiseti* st.2, it was suppressed under fungicide treatment, suggesting treatment-dependent transcriptional modulation, in agreement with previous reports where PR5 expression varied between resistant and susceptible genotypes [79]. PR8, a chitinase gene, was strongly repressed in *F. equiseti* st.1 under fungicide but remained more stable in *F. equiseti* st.2 [77]. Collectively, these findings indicate that ZnO-NPs activate stronger and broader defense gene expression than fungicide, particularly for PPO and PR2, suggesting that nanoparticles may enhance fungal stress responses and resistance mechanisms. These observations align with earlier studies highlighting the importance of early defense signaling and PR gene activation in resistance against *Fusarium* sp [80].To the best of our knowledge, only a few studies have specifically investigated the use of biosynthesized ZnO-NPs as antifungal agents against *F. equiseti.* In our study, biosynthesized ZnO-NPs from *Trichoderma asperellum* exhibited maximum inhibition rates of 72.41 ± 0.01 and 73.41 ± 1.16% against *F. equiseti* st.1 and st.2 at a concentration of 5 mg/mL. A comparable report from Nepal demonstrated that tea leaf-mediated ZnO-NPs achieved up to 85% inhibition of *F. equiseti* at 1.2 mg/mL [40], suggesting that plant-based synthesis can generate effective antifungal nanoparticles, although generally requiring higher concentrations compared to fungal mediated synthesis.

In comparison with *Trichoderma* based systems, ZnO-NPs synthesized by *T. harzianum* demonstrated 72.5% inhibition of *F. oxysporum* at 1.0 mg/mL [78], while another report achieved a similar 72% inhibition at only 5 µg/mL [81]. These findings indicate that nanoparticle efficacy depends not only on dosage but also on the targeted fungal species and the biosynthetic route employed. Morphologically, *T. harzianum* derived ZnO-NPs were reported to have large average sizes 517 nm [45], whereas *T. asperellum* yielded smaller and more uniform structures [82]. In our case, SEM analysis showed ZnO-NPs ranging from 13–19 nm, which is closer to the smaller particle morphologies described in other fungal synthesis studies, highlighting the role of fungal metabolites in modulating particle size, capping, and stability.

Beyond *Trichoderma*, algal derived ZnO-NPs inhibited *F. oxysporum* and *R. solani* by 63.4–88.9% at 100 mg/mL [83], while *Penicillium* based ZnO-NPs achieved nearly complete inhibition 100% of *F. oxysporum* at 1000 µg/mL [43]. Collectively, these comparative results suggest that the biosynthetic origin strongly influences nanoparticle properties such as particle size, zeta potential, and aggregation behavior, which in turn determine antifungal efficiency. Our findings therefore contribute novel insights into the use of *Trichoderma asperellum* mediated ZnO-NPs against *F. equiseti*, supporting their potential as eco-friendly and effective antifungal agents.

## Conclusion

This study demonstrates the successful green synthesis of ZnO-NPs using *Trichoderma asperellum* filtrate, resulting in stable, crystalline, and uniformly sized nanoparticles with potent antifungal activity against *Fusarium equiseti*. The myco synthesized ZnO-NPs achieved inhibition rates of 72.41 ± 0.01% and 73.41 ± 1.16% against the two tested strains, showing efficacy comparable to that of the commercial fungicide. GC–MS profiling revealed that ZnO-NPs induced notable metabolic reprogramming, including the emergence of unique stress-associated metabolites, while qRT–PCR analysis demonstrated selective upregulation of defense-related genes such as PR2, PPO, and PR5. These integrated biochemical and molecular responses suggest that ZnO-NPs suppress F. equiseti not only through direct structural damage but also by modulating host stress and defense pathways. Owing to their eco-friendly synthesis, biocompatibility, and strong antifungal efficacy, mycogenic ZnO-NPs represent a promising and sustainable alternative to conventional chemical fungicides in integrated crop disease management.

## Supplementary Information

Below is the link to the electronic supplementary material.

Supplementary Material 1

## Data Availability

Data is provided within the manuscript or supplementary information files.
