# Supplementary material for: Green mycosynthesis of ZnO nanoparticles enhances antifungal defense against Fusarium equiseti through metabolic and gene expression modulation
Source: Discov Nano. 2026 Jan 30;21(1):19. doi: 10.1186/s11671-026-04429-5 (PMC12858691; doi:10.1186/s11671-026-04429-5)
Supplement: Supplementary file 1 — Supplementary Material 1 [file 11671_2026_4429_MOESM1_ESM.doc]

**Supplementary information for**

**Green Mycosynthesis of ZnO Nanoparticles Enhances Antifungal Defense Against Fusarium equiseti Through Metabolic and Gene Expression Modulation**

EL‑Sayed M. El‑Morsy^a^, Yomna S. Elmalahy^a^* and Elsayed E. Hafez^b^

*^a^ Botany and Microbiology Department, Faculty of Science, Damietta University, New Damietta 34517, Egypt*

*^b^ Plant protection and Bimolecular diagnosis department, (Arid Lands Cultivation Research Institute), City of Scientific Research and Technological Applications, New Borg El-Arab, Egypt.*


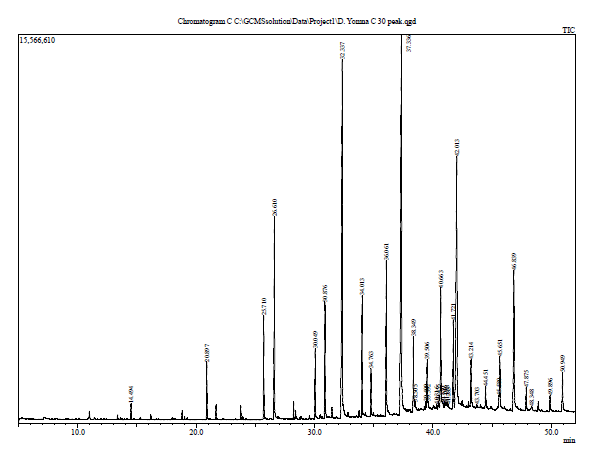


Fig.S1.GC-MS spectra for *Fusarium equisiti* st.1


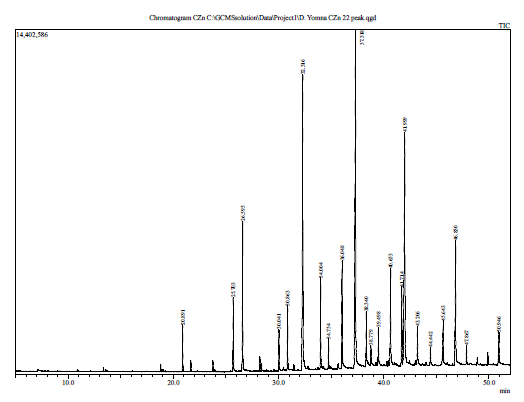
 Fig.S2. GC-MS spectra for *Fusarium equisiti* st.1 when treatment with ZnO-NPs


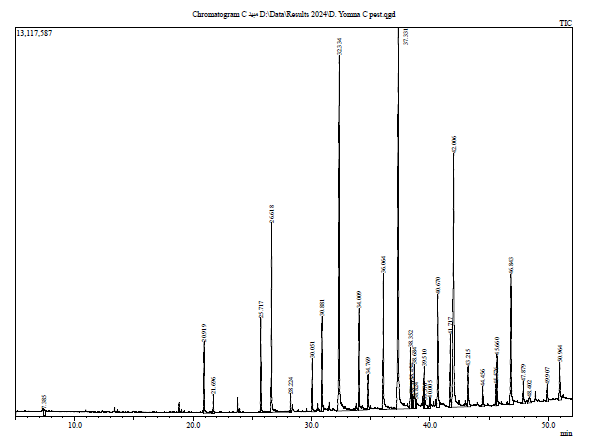
 Fig.S3. GC-MS spectra for *Fusarium equisiti* st.1 when treatment with fungicide


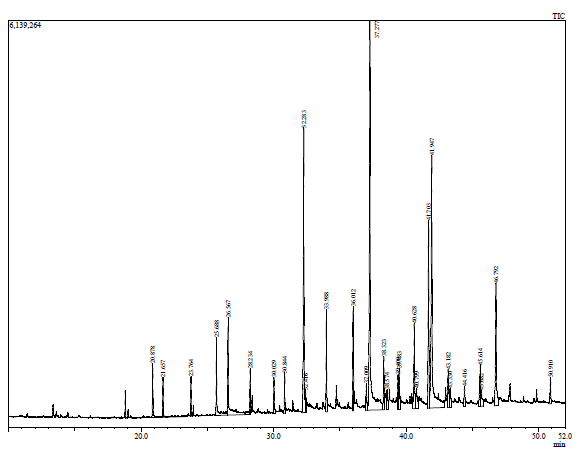
Fig.S4. GC-MS spectra for *Fusarium equisiti* st.2


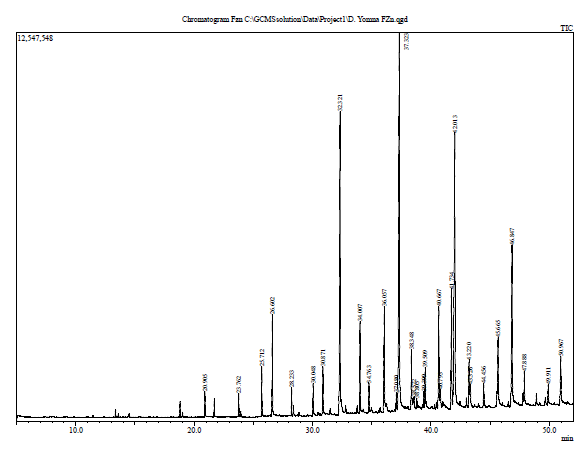


Fig.S5. GC-MS spectra for *Fusarium equisiti* st.2 when treatment with ZnO-NPs


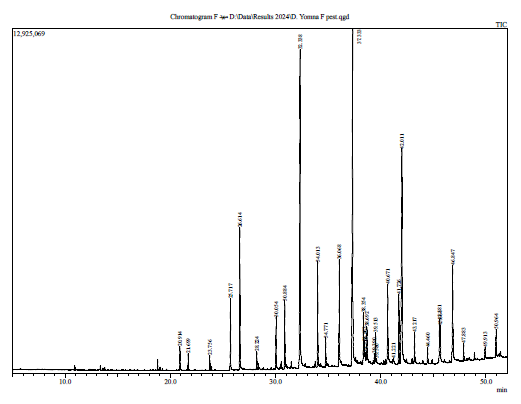
 GC-Fig.S6. GC-MS spectra for *Fusarium equisiti* st.2 when treatment with fungicide
